# New 3D Spiral Microfluidic Platform Tested for Fe_3_O_4_@SA Nanoparticle Synthesis

**DOI:** 10.3390/molecules30142896

**Published:** 2025-07-08

**Authors:** Elena-Theodora Moldoveanu, Adelina-Gabriela Niculescu, Dana-Ionela Tudorache (Trifa), Alina Moroșan, Alexandra-Cătălina Bîrcă, Bogdan-Ștefan Vasile, Ariana Hudita, Dan-Eduard Mihaiescu, Tony Hadibarata, Alexandru-Mihai Grumezescu

**Affiliations:** 1 Department of Science and Engineering of Oxide Materials and Nanomaterials, National University of Science and Technology POLITEHNICA Bucharest, 011061 Bucharest, Romania; elena.moldoveanu99@upb.ro (E.-T.M.); alexandra.birca@upb.ro (A.-C.B.); tony.hadibarata@upb.ro (T.H.); grumezescu@yahoo.com (A.-M.G.); 2 Research Institute of the University of Bucharest—ICUB, University of Bucharest, 050657 Bucharest, Romania; ariana.hudita@unibuc.ro; 3 Department of Organic Chemistry, National University of Science and Technology POLITEHNICA Bucharest, 011061 Bucharest, Romaniadanedmih@gmail.com (D.-E.M.); 4 Research Center for Advanced Materials, Products and Processes, National University of Science and Technology POLITEHNICA Bucharest, 060042 Bucharest, Romania; bogdan.vasile@upb.ro; 5 National Research Center for Micro and Nanomaterials, National University of Science and Technology POLITEHNICA Bucharest, 060042 Bucharest, Romania; 6 Department of Biochemistry and Molecular Biology, University of Bucharest, 91-95 Splaiul Independentei Street, 050095 Bucharest, Romania; 7 Environmental Engineering Program, Faculty of Engineering and Science, Curtin University Malaysia, CDT 250, Miri 98009, Malaysia

**Keywords:** microfluidic technology, 3D microfluidics, spiral microfluidic platform, nanoparticle synthesis, lab-on-a-chip, magnetite, salicylic acid, on-chip functionalization

## Abstract

Due to the need for reproducible, scalable, and environmentally friendly nanomaterial synthesis methods, an increasing amount of scientific interest revolves around microfluidic technologies. In this context, the present paper proposes a new three-dimensional (3D) spiral microfluidic platform designed and tested for the simultaneous synthesis and surface functionalization of magnetite (Fe_3_O_4_) nanoparticles with salicylic acid (SA). The microreactor was fabricated from overlaid polymethylmethacrylate (PMMA) sheets and assembled into a compact, reusable chip architecture, allowing continuous reagent mixing and enhanced hydrodynamic control. The performed physicochemical analyses confirmed that on-chip synthesized Fe_3_O_4_@SA NPs exhibit crystallinity, a uniform spherical morphology, a narrow size distribution, excellent colloidal stability, and successful surface functionalization. In vitro cytotoxicity assays using MRC-5 lung fibroblasts and HaCaT keratinocytes revealed a concentration-dependent response, identifying a safe dose range below 610 µg/mL. The integrated design, efficient synthesis, and favorable biocompatibility profile position this 3D microfluidic platform as a promising tool for scalable nanomaterial production in biomedical and environmental applications.

## 1. Introduction

Nanotechnology represents a revolutionary area of material science, gaining substantial interest as researchers acknowledged the critical role of particle size in determining physicochemical properties and highlighted their implications in advancing various domains (e.g., biomedicine, electronics, and environmental remediation) [1,2,3,4]. Considering their unique features and wide applicability, numerous synthesis methods have been developed to fabricate nanomaterials, with many of them being appropriate for obtaining magnetite nanoparticles (Fe_3_O_4_ NPs). Among the various synthesis strategies, co-precipitation represents one of the most commonly employed methods for producing Fe_3_O_4_ NPs due to its simplicity and cost-effectiveness. However, it often suffers from poor control over particle size and morphology and batch-to-batch variability [5,6]. Mechanochemical synthesis has been explored as a solvent-free, energy-efficient method for magnetite production; yet, it requires specialized milling equipment and typically leads to a broad particle size distribution [7]. More recently, hydrogen-wet reduction has emerged as a promising route, particularly for large-scale magnetite nanopowder preparation, though the method involves high-temperature processing and safety concerns due to hydrogen gas handling [8].

In more general terms, conventional synthesis methods are usually classified as top-down or bottom-up techniques, each having its advantages and disadvantages. The top-down approach involves reducing bulk materials to nanoscale dimensions, often resulting in oxidation susceptibility, agglomeration, and crystallographic defects. Conversely, the bottom-up approach, primarily based on wet chemical synthesis, offers advantages in adaptability, cost-effectiveness, and scalability, but is limited by challenges in reaction kinetics control and reproducibility [9,10,11].

From the point of view of the involved processes, top-down and bottom-up fabrication methods can be further divided into physical, chemical, and biological methods [3,12]. Though widely used, physical and chemical methods often raise environmental concerns due to the release of toxic compounds and their significant energy demands. Additionally, these methods require large, expensive equipment, involve time-consuming and complex procedures, and frequently suffer from poor reproducibility. The resulting nanomaterials may exhibit broad size distributions (high polydispersity), potential contamination, and suboptimal surface structures, compromising their functional properties and further applicability [3,13,14,15,16]. On the other hand, biological synthesis techniques (often called “green synthesis”) represent a more sustainable and environmentally friendly alternative, typically employing biomass or biological systems. However, these methods require further in-depth studies to understand how natural active compounds influence the resulting nanoparticles’ polydispersity, stability, biocompatibility, and biodistribution [17,18,19,20].

Overall, the limitations of conventional synthesis methods highlight the need for alternative synthesis strategies that ensure better control over nanoparticle characteristics while minimizing the environmental impact [19,21]. In recent years, microfluidic technology has emerged as a promising platform for nanomaterial synthesis, offering superior control over reaction conditions through precise fluid manipulation at the microscale level [22,23]. Microfluidic reactors outperform conventional batch synthesis by ensuring homogeneous mixing, rapid heat transfer, and efficient reagent utilization, improving monodispersity, reproducibility, and product quality [3,24]. Furthermore, their miniaturized design facilitates process automation, reduces material waste, and allows for the real-time optimization of synthesis parameters [25]. Compared to traditional methods, microfluidic platforms enable fine-tuned control over nanoparticle size, shape, and composition through adjustments in the channel geometries, flow rates, and reagent concentrations [6,25,26].

The hydrodynamic behavior of microfluidic systems plays a crucial role in nanoparticle formation, with channel geometry and mixing efficiency significantly impacting synthesis outcomes. Researchers have developed advanced microfluidic designs capable of optimizing nanoparticle properties by modifying structural features such as channel curvature, cross-section, and mixing patterns [25,27,28,29,30]. Additionally, microfluidic synthesis minimizes reaction times and enhances heat and mass transfer, preventing temperature gradients that typically affect batch synthesis processes [31,32].

Usually, 2D microreactors can have planar mixing systems varying from common T-type or Y-type junctions to more complex configurations (e.g., spirals, S-shaped channels, coflowing junctions, hydrodynamic flow focusing, staggered herringbone structure, or combined geometries) [25,33]. Nonetheless, such systems may exhibit certain disadvantages related to the utilization of narrow channels, which, combined with a laminar flow regime, predispose the chips to clogging and a limited production rate. Moreover, the mixing efficiency in planar microfluidics may not be adequate over short channel lengths, leading to the formation of non-uniform products [3,34,35].

To address the limitations of 2D systems and integrate functionality in a single step, we developed a new 3D spiral microfluidic platform. To our knowledge, there is limited evidence of the integration of 3D flow dynamics with on-chip surface functionalization, which is critical for biomedical-grade nanoparticle production.

Emerging microfluidic platforms have tackled the possibility of introducing a third dimension for improving reagent homogenization and creating 3D flow patterns within microreactors [36]. Three-dimensional microfluidic platforms offer substantial advantages over conventional planar micromixers by leveraging the additional spatial dimension to enhance the mixing efficiency, reaction kinetics, and overall process control. Incorporating the z-direction flow dynamics can significantly increase fluid contact times and surface interactions, intensifying the mixing and reducing the channel length requirements, surpassing the two-dimensional design limitations [34]. This advanced architecture enables superior reaction homogeneity, improved mass transfer, and more precise control over particle synthesis parameters such as size, morphology, and composition [37]. Moreover, 3D microfluidic reactors facilitate scalable, continuous-flow nanoparticle production with minimized reagent consumption, reduced waste generation, and lower energy demands, aligning with sustainable and eco-friendly synthesis practices [38]. Despite these advantages, challenges remain in the large-scale fabrication and industrial translation of 3D micromixers due to the complexity of their structural design and the need for optimized manufacturing processes [39].

As microfluidics continues to evolve, interdisciplinary research in materials science, engineering, and biomedicine is expected to drive significant chip design and integration advancements, enabling scalable and sustainable nanomaterial synthesis [25,30].

Given the increasing demand for scalable, eco-friendly, and multifunctional synthesis routes, the present study explores the potential of three-dimensional microfluidic technologies in overcoming the limitations of conventional nanomaterial synthesis, emphasizing their role in achieving high-quality, reproducible, and environmentally sustainable nanomaterials. Specifically, a new design is proposed for a 3D microfluidic platform capable of the simultaneous synthesis of magnetite (Fe_3_O_4_) nanoparticles and their surface functionalization with salicylic acid (SA) toward creating fine-tuned nanosystems for advanced applications.

The motivation behind designing a novel 3D spiral microfluidic platform stems from the need to overcome the limitations in mixing efficiency and integration capabilities observed in conventional 2D microfluidic chips. We herein introduce a modular, laser-fabricated 3D spiral platform capable of both synthesizing magnetite nanoparticles and conjugating salicylic acid in a single continuous step, under controlled hydrodynamic conditions. Salicylic acid was chosen as the coating agent due to its ability to improve water dispersibility and long-term stability [37,40], as well as recognized antioxidant and anti-inflammatory properties [41,42], which are valuable for biomedical applications such as wound healing, antimicrobial systems, or drug delivery. This study also focuses on establishing the biocompatibility of the on-chip synthesized materials using healthy human cell lines as a baseline. Therefore, these findings set the groundwork for further exploration of the Fe_3_O_4_@SA nanoparticle behavior in complex biological models or environmental setups.

## 2. Results

To evaluate the potential of the newly developed microfluidic platform for nanoparticle synthesis, the chip was tested for Fe_3_O_4_@SA production. The obtained materials were further analyzed from the morpho-structural and biological perspectives to establish the essential characteristics and verify the success of the on-chip simultaneous synthesis and surface functionalization.

**Figure 1:**
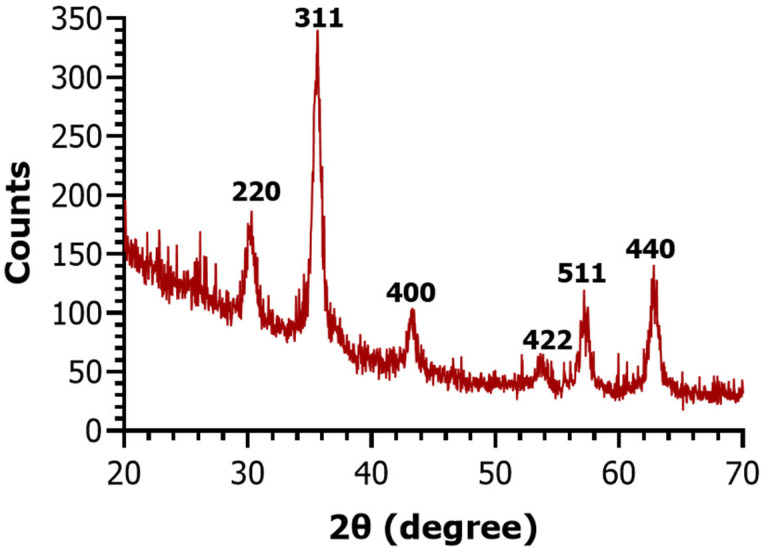
X-ray diffractogram of Fe_3_O_4_@SA nanoparticles.

Based on XRD analysis, the crystalline phases from the sample were identified, with the diffraction pattern displayed in Figure 1. Sharp diffraction peaks were observed at 2θ angles 30.18°, 35.42°, 43.22°, 53.62°, 57.18°, and 62.85°, which are associated with the (220), (311), (400), (422), (511), and (440) diffraction planes, respectively. The identified pattern is characteristic of the cubic spinel crystallographic system of magnetite, revealing it as the only phase from the sample [37,38]. These results confirm the crystallinity and purity of the nanomaterial obtained on the microfluidic platform.

The sample was further analyzed using the DLS technique to gather data regarding the hydrodynamic diameter, the polydispersity index, and the zeta potential. The DLS results (Table 1) demonstrate nanoscale dimensions for the analyzed material, confirming that the proposed synthesis device results in the formation of nanomaterials with a narrow size distribution. The registered zeta potential has a remarkable absolute value (i.e., ~78.5 mV) that reflects the excellent colloidal stability of the Fe_3_O_4_@SA dispersion and its non-sedimentation properties.

**Figure 2:**
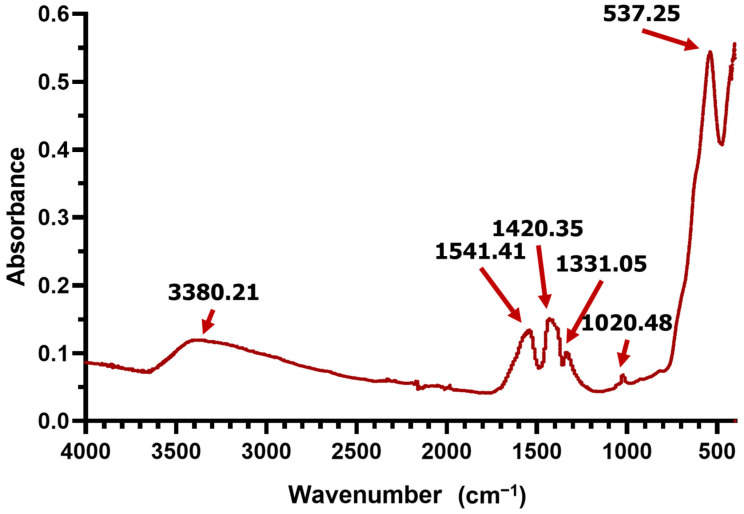
FT-IR spectrum of Fe_3_O_4_@SA nanoparticles.

An FT-IR analysis was performed to gain information on the compositional groups present in the samples (Figure 2). The FT-IR spectrum of the Fe_3_O_4_@SA nanocomposite synthesized via a microfluidic platform confirms the presence of both magnetite and the organic shell. The characteristic Fe–O stretching vibration of Fe_3_O_4_ appears as a strong absorption band at ~537 cm^−1^, highlighting the successful formation of the magnetic core. The broad absorption in the 3200–3500 cm^−1^ range is attributed to O–H stretching, confirming the retention of hydroxyl groups. The peaks observed in the 1550–1300 cm^−1^ region correspond to the C=C stretching of the aromatic ring present in the structure of the functionalization agent. The observed red shift in the C=C stretching vibrations of the aromatic ring (from the expected ~1600–1400 cm^−1^ region to 1541, 1420, and 1331 cm^−1^) is likely due to the coordination interactions between salicylic acid and the Fe_3_O_4_ surface. When salicylic acid binds to the magnetite core, its carboxylate and hydroxyl groups can chelate Fe^3+^ ions, altering the electron density distribution within the aromatic system, and leading to a lower wavenumber in the corresponding FT-IR peaks [43].

**Figure 3:**
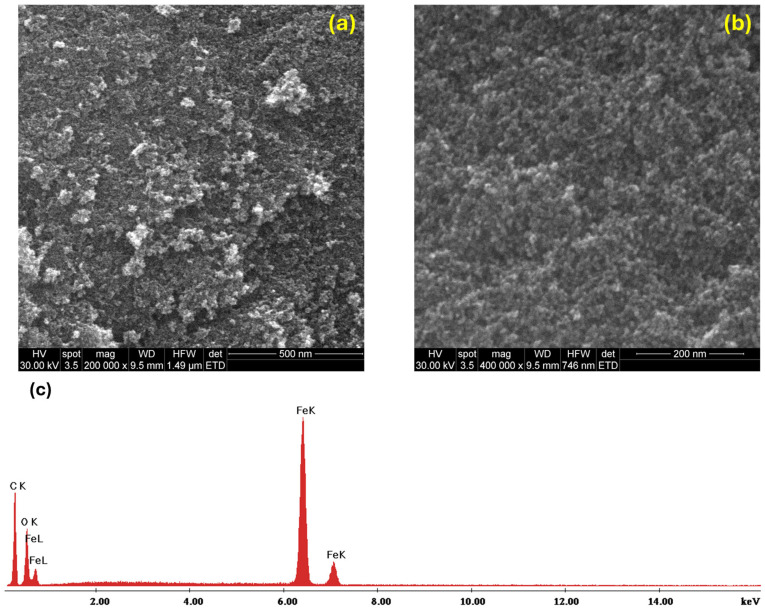
SEM micrographs at (**a**) 200,000× and (**b**) 400,000× magnification and (**c**) EDS analysis of Fe_3_O_4_@SA nanoparticles.

The morpho-structural assessment enabled by SEM (Figure 3a,b) revealed a uniformity within the Fe_3_O_4_@SA sample, emphasizing the presence of nanodimensional particles with a reduced aggregation tendency. Moreover, the micrographs were complemented by an EDS analysis (Figure 3c), with the spectrum evidencing the elemental composition of the synthesized material. Specifically, peaks for iron and oxygen have been obtained, which reconfirm the formation of iron oxide.

**Figure 4:**
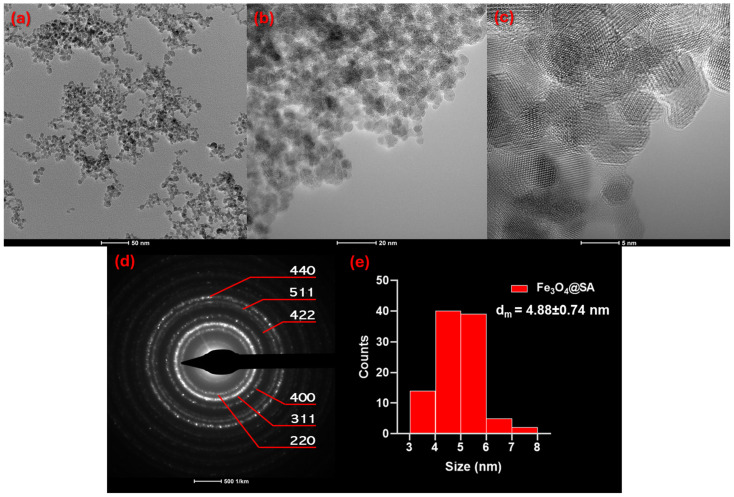
(**a**,**b**) TEM and (**c**) HR-TEM micrographs, (**d**) SAED pattern with the corresponding Miller indices, and (**e**) size distribution of Fe_3_O_4_@SA nanoparticles.

TEM micrographs (Figure 4a–c) allowed a better visualization of the nanoparticles’ morphology, demonstrating their spherical shape, uniform ultrasmall dimensions, and reduced agglomeration tendency. According to the size distribution of the nanoparticles displayed in Figure 4e, Fe_3_O_4_@SA nanostructures have a monomodal distribution, with a calculated average particle size of 4.88 ± 0.74 nm. These values are several-fold smaller than the measurements obtained with the DLS analysis. The discrepancy between the hydrodynamic diameter measured by DLS (~146 nm) and the core size observed in TEM (~5 nm) is attributable to the fundamental differences between these characterization techniques. Specifically, DLS reflects the hydrodynamic diameter, encompassing the magnetic core, the organic salicylic acid surface coating, the solvation layer, and dynamic aggregates in aqueous dispersion. At the same time, TEM measurements only reveal the solid core dimensions under vacuum [44]. These factors commonly yield larger hydrodynamic diameters and are consistent with the literature reports for surface-functionalized iron oxide nanoparticles exhibiting high surface activity and colloidal stabilization [45,46,47,48].

Moreover, the SAED pattern (Figure 4d) shows six evident concentric rings that confirm sample crystallinity, being in agreement with the ring structure of magnetite crystallized in the inverse spinel cubic structure (space group: Fd̅3m). Specifically, diffraction rings have been identified for the face-centered cubic lattice corresponding to the (220), (311), (400), (422), (511), and (440) planes. This pattern also matches XRD determinations, confirming magnetite as a single phase.

**Figure 5:**
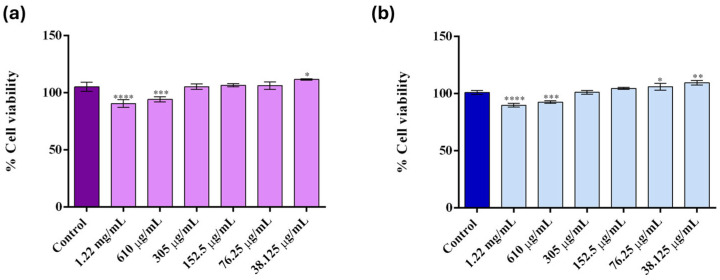
Graphical representation of (**a**) human lung fibroblasts MRC-5 and (**b**) human keratinocytes HaCaT cell viability after 48 h of treatment with various concentrations of Fe_3_O_4_@SA nanoparticles. The represented data are the mean values of three independent experiments ± S.D. (* *p* ≤ 0.05; ** *p* ≤ 0.01; *** *p* ≤ 0.001; **** *p* ≤ 0.0001 sample vs. control).

To evaluate the metabolic activity and overall viability of human lung fibroblasts (MRC-5) and human keratinocytes (HaCaT) exposed to various concentrations of Fe_3_O_4_@SA nanoparticles synthesized via the microfluidic chip, the MTT assay was performed following 48 h of exposure to treatment (Figure 5). These cell lines were chosen to provide a baseline cytocompatibility profile for future biomedical applications.

In MRC-5 cell cultures (Figure 5a), high concentrations of Fe_3_O_4_@SA nanoparticles (1.22 mg/mL and 610 μg/mL) induced a statistically significant decrease in cell viability compared to the untreated control, suggesting a concentration-dependent cytotoxic effect on lung fibroblasts. However, the negative impact of these concentrations on cell viability was moderate, with the viability reduced by only ±10.32% and ±7.6%, respectively, compared with the untreated control. In contrast, lower concentrations of Fe_3_O_4_@SA nanoparticles did not significantly impact cell viability as compared with the untreated control. Moreover, in some cases, the metabolic activity of human fibroblasts appeared slightly elevated compared to the control. A similar pattern of cell viability under treatment was also observed for HaCaT cells (Figure 5b). The same two concentrations (1.22 mg/mL and 610 μg/mL) triggered a statistically significant decrease in cell viability compared to the untreated control, highlighting a comparable cytotoxic response in keratinocytes. At concentrations below 610 μg/mL, no cytotoxic effects were observed in HaCaT cells, with viability levels remaining comparable or slightly enhanced relative to the untreated control.

To further investigate the cytotoxic profile of Fe_3_O_4_@SA nanoparticles, the Live/Dead assay was performed after 48 h of treatment to complement the MTT assay and enable a qualitative visualization of both live and dead cells following treatment exposure.

**Figure 6:**
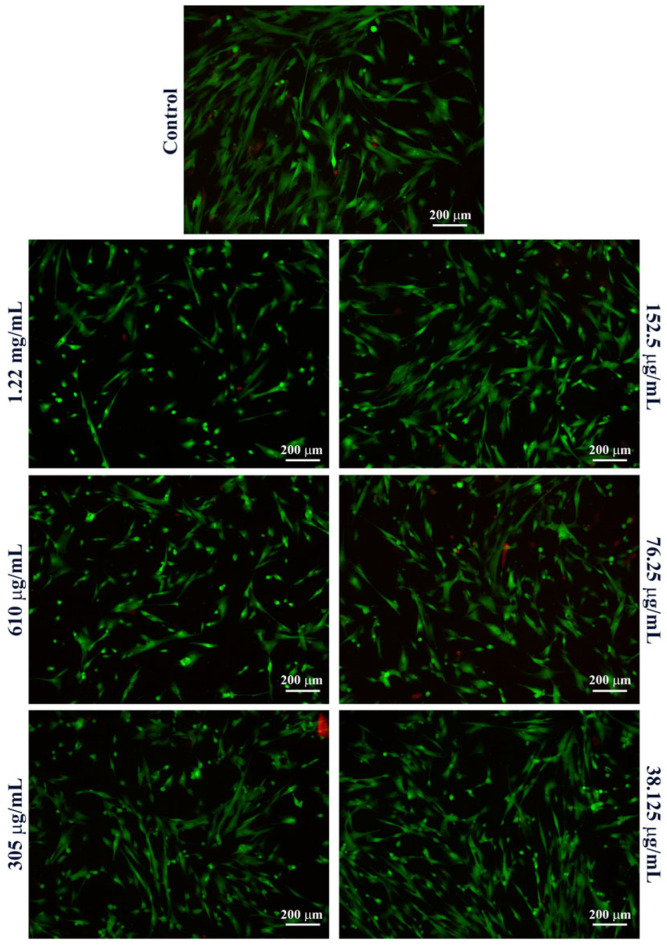
Fluorescence micrographs of live (green) and dead (red) human lung fibroblasts after 48 h of treatment with various concentrations of Fe_3_O_4_@SA nanoparticles (10× magnification).

In MRC-5 cell cultures (Figure 6), a clear predominance of green fluorescent cells (viable) was observed across all treatment groups, indicating a high overall cell viability. Interestingly, even at high concentrations of Fe_3_O_4_@SA nanoparticles (1.22 mg/mL and 610 μg/mL), only a limited presence of red-stained cells (non-viable) was detected. However, at these concentrations, a noticeable proportion of cells appeared rounded, indicating cytoskeletal or adhesion-related changes compared to the untreated control, where cells exhibited their typical elongated, spindle-shaped fibroblast morphology. Therefore, despite the lack of red-stained cells under these conditions, these alterations suggest cellular stress following exposure to high concentrations of Fe_3_O_4_@SA nanoparticles.

**Figure 7:**
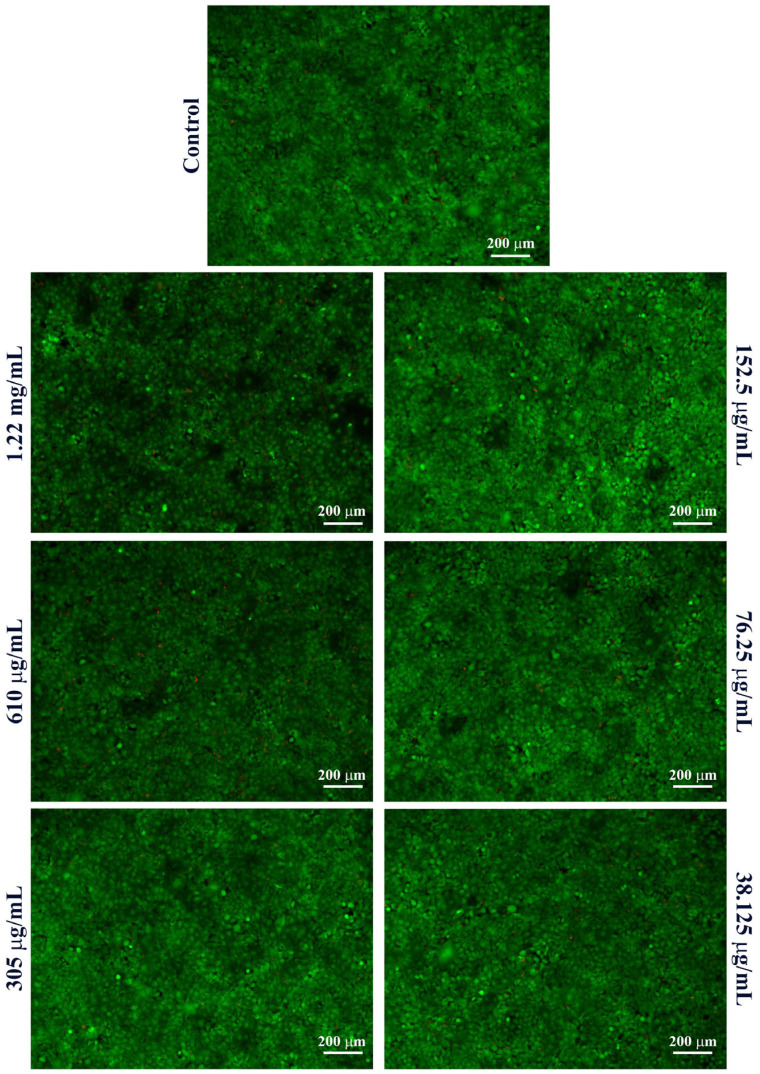
Fluorescence micrographs of live (green) and dead (red) human keratinocytes after 48 h of treatment with various concentrations of Fe_3_O_4_@SA nanoparticles (10× magnification).

In HaCaT cell cultures (Figure 7), the obtained results also highlighted a high overall cell viability under all tested conditions, with intense green fluorescent cells present, similar to the untreated control. However, at high concentrations, a marked decrease in cell density was observed at the highest concentrations tested, particularly at 1.22 mg/mL. This decrease in adherent viable cells confirms the MTT assay results, suggesting that high concentrations of Fe_3_O_4_@SA nanoparticles impact keratinocyte survival and adhesion. The green fluorescent signal was high at lower concentrations, confirming the lack of cytotoxic effects of the Fe_3_O_4_@SA nanoparticles under 610 μg/mL.

## 3. Discussion

The development of efficient and scalable methods for nanoparticle synthesis remains a critical challenge in nanotechnology, particularly when high uniformity, stability, and functional versatility are desired. Given the plethora of advantages microfluidic reactors offer, numerous studies have explored such devices for synthesizing various materials. Geometries involving different types of mixing have been utilized to obtain nanofibers [49], microcapsules [50,51,52,53], microdroplets [54,55], double emulsion droplets [56,57], nanogels [58], porous films [59], microparticles [60,61,62], and nanoparticles [63,64,65]. However, most of these studies have utilized two-dimensional geometries.

In this context, this paper proposes the utilization of a newly designed 3D platform that can overcome the well-known associated limitations of its 2D counterpart microfluidic chips. Other 3D microfluidic platforms reported in the literature have focused on the synthesis of core-shell droplets [66], optically pure γ-lactones [67], polymeric nanoparticles [68], zinc oxide nanoparticles [69], and active pharmaceutical ingredients [70]. Additionally, the herein-developed 3D spiral microreactor expands the possibilities of nanomaterial synthesis, enabling the fabrication of uniform quasi-spherical magnetite nanoparticles in a short time and with high productivity.

Compared to conventional batch synthesis, which often suffers from poor reproducibility, broad size distributions, and high reagent consumption, the 3D spiral microfluidic platform offers enhanced control over reaction parameters, continuous-flow operation, and high product uniformity [28,71,72]. Introducing a third spatial dimension improves mixing efficiency and reaction homogeneity beyond what planar 2D microfluidic systems typically allow, particularly in laminar flow regimes [71]. The modular, laser-cut PMMA design also enables chip reusability and scalable, inexpensive fabrication [73,74,75]. However, 3D microfluidic systems can be more complex to design and fabricate than standard 2D geometries or batch setups, and may require the fine-tuning of flow dynamics to avoid backpressure or channel misalignment [28,73]. For better clarity, the advantages and limitations of conventional methods and microfluidic approaches are summarized in Table 2.

A major advantage of the presented microfluidic platform lies in its modular, layer-by-layer design, which facilitates both the reproducibility and scalability of nanoparticle synthesis. Using laser-cut PMMA layers enables a precise and repeatable channel architecture, ensuring uniform fluid dynamics and consistent nanoparticle characteristics across multiple fabrication batches. Such modular fabrication strategies can significantly reduce the variability in microfluidic operations compared to soft-lithography-based devices, particularly when implemented using automated or computer-aided design tools [34]. Moreover, controlling flow rates in the present spiral design offers a viable route toward process intensification, optimizing the material throughput while maintaining nanoscale control. Previous studies have demonstrated that scaling up microfluidic systems through parallelization or multilayer stacking can effectively increase production rates without compromising nanoparticle quality [9,27]. These attributes make the current platform a valuable tool for laboratory-scale synthesis and a promising candidate for translation into continuous-flow, industrial-scale nanomaterial production, especially in biomedical and environmental applications where batch-to-batch consistency is critical.

The proposed multi-layered platform supported the simultaneous formation and functionalization of surface-modified iron oxide nanoparticles with salicylic acid. The incorporation of vertical mixing dynamics within a spiral geometry enhances reaction uniformity and enables the efficient conjugation of functional molecules during particle formation. The results demonstrate that the unique multi-layered spiral configuration enhances the mixing efficiency and reaction control, forming well-defined, monodisperse nanostructures with excellent colloidal stability.

In addition to the desirable physicochemical properties, promising results have also been obtained through biological characterization. The cytotoxic potential of the Fe_3_O_4_@SA nanoparticles synthesized via the microfluidic chip was investigated in vitro using two human-derived cell lines: lung fibroblasts MRC-5 and HaCaT keratinocytes. Both MTT and Live/Dead assays revealed a concentration-dependent cytotoxicity pattern in both cellular models, with a statistically significant decrease in cell viability observed at concentrations ≥ 610 μg/mL compared to the untreated control. Notably, the decrease in cell viability was moderate, suggesting low cytotoxic effects without triggering extensive cell death. At sub-toxic doses, Fe_3_O_4_@SA not only maintained but, in some cases, slightly increased cell viability, suggesting a concentration threshold below which these particles may exhibit cytoprotective or proliferative effects. These findings can be attributed to the presence of SA, known for its anti-inflammatory and antioxidant properties, which could contribute to enhanced metabolic activity [76]. In summary, all the results confirmed the concentration-dependent cytotoxic profile of the tested nanoparticles and allowed for the identification of a non-toxic working range that enables a safety profile of Fe_3_O_4_@SA nanoparticles. Our findings are consistent with the previous studies reporting the good biocompatibility of Fe_3_O_4_ nanoparticles at low doses, either simple or functionalized with bioactive compounds [77,78,79].

By fine-tuning the dosage and selecting the non-toxic concentration range, Fe_3_O_4_@SA NPs show great promise for further biomedical applications where maintaining cellular cytocompatibility is a crucial feature. The synthesized Fe_3_O_4_@SA nanoparticles exhibited a core diameter of 4.88 ± 0.74 nm (TEM) and a hydrodynamic diameter of 146.3 nm (DLS) with a PDI of 0.2, indicating a relatively narrow size distribution and good colloidal uniformity. These values are comparable to or better than those reported for other iron oxide-based nanoparticles functionalized for biomedical use. For example, Foglia et al. [77] have reported the synthesis of sub-5 nm silica-coated magnetite particles (DLS ≈ 30–50 nm) with excellent biocompatibility in Caco-2 cells at concentrations between 10 and 100 μg/mL. Differently, Putz and colleagues [80] investigated the biocompatibility of hybrid silica-PVA–iron oxide nanocomposites (<20 nm core size), reporting a good HaCaT cell viability up to a concentration of 100 μg/mL. Moreover, reviews of Fe_3_O_4_-based nanoparticle applications commonly report hydrodynamic sizes between 20–200 nm with PDI < 0.3 and biocompatibility at moderate concentrations [81,82]. Our Fe_3_O_4_@SA particles maintained >90% viability in normal human cell lines (i.e., MRC-5, HaCaT) up to 610 µg/mL, underscoring an advantageous biocompatibility window in comparison.

Therefore, the obtained nanomaterials exhibit desirable properties for further advanced applications, benefiting from the high zeta potential, narrow size distribution, magnetic manipulation, and bioactivity. These features enable the future exploration of on-chip synthesized Fe_3_O_4_@SA NPs for drug delivery, biosensing, or environmental remediation [23,37,38,39,83], encouraging more in-depth studies for applicability confirmation. For instance, future studies should extend this work to more complex biological environments, including 3D tissue constructs and animal models, to confirm biodistribution, clearance, and therapeutic potential. Furthermore, exploring the platform’s adaptability for drug loading and controlled release would expand its biomedical relevance, particularly in nanocarrier development.

What sets this platform apart is its ability to simultaneously synthesize and surface-functionalize nanoparticles within a single 3D chip—an achievement not commonly reported in comparable systems. This dual-action capability simplifies downstream processing and opens the door to tailored surface chemistries for targeted applications. From a sustainability standpoint, the system aligns well with the principles of green nanotechnology by minimizing reagent waste, energy consumption, and solvent use—attributes that are crucial for eco-conscious nanomaterial production [20,21]. While this study demonstrates a proof of concept for the synthesis and functionalization process, the chip’s modular architecture and controlled flow parameters support high reproducibility. In future work, we plan to conduct batch-to-batch reproducibility assessments and long-term operational stability studies to confirm the process consistency quantitatively. Thus, as microfluidic technology continues to evolve, platforms such as this custom-made spiral 3D microreactor are poised to bridge the gap between laboratory innovation and industrial-scale nanomanufacturing.

## 4. Materials and Methods

### 4.1. Materials

For microfluidic platform fabrication and assembly, polymethylmethacrylate (PMMA) sheets of 2 mm width, commercial bicomponent epoxy adhesive (“Epoxy Universal”, Bison International B.V., Rotterdam, The Netherlands), 8 mm polypropylene tubing, and push-fit connectors were utilized.

For nanoparticle synthesis, we used reagents of analytical purity, purchased from Sigma Aldrich Merck (Darmstadt, Germany)—ferric chloride (FeCl_3_) and iron sulfate heptahydrate (FeSO_4_·7H_2_O), Lach-Ner (Tovarni, Czech Republic)—sodium hydroxide (NaOH), Emsure Merck Millipore (Darmstadt, Germany)—acetic acid, and ATOCHIM PROD (Bucharest, Romania)—salicylic acid. Ultrapure water (resistivity 18.2 MΩ·cm, TOC < 5 ppb), obtained from a Milli-Q purification system (Millipore, Burlington, MA, USA), was used in all synthesis and washing steps.

For the in vitro biological assays, the cell cultures were purchased from American Type Cell Culture Collection (ATCC, Manassas, VA, USA), while most of the reagents required for cell maintenance and biological assays were purchased from Sigma Aldrich Merck (Darmstadt, Germany), except for the fetal bovine serum (FBS) and the LIVE/DEAD Viability/Cytotoxicity Kit, which were purchased from ThermoFisher Scientific (Waltham, MA, USA).

### 4.2. Microfluidic Platform Design

**Figure 8:**
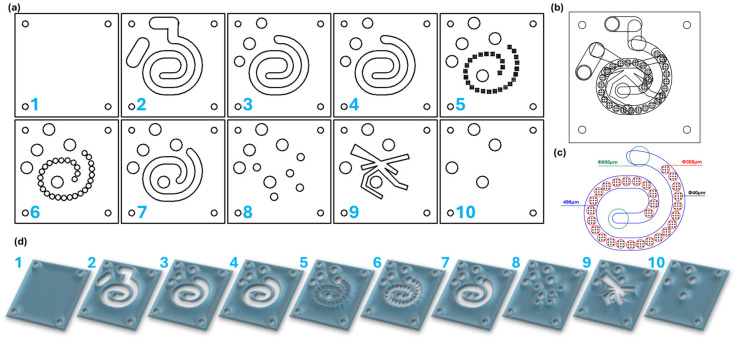
Microfluidic platform design: (**a**) schematic 2D representation of the microfluidic platform individual layers; (**b**) overlapped 2D layer design; (**c**) overlayed reaction area and its dimensions; and (**d**) 3D representation of the microfluidic platform individual layers.

RDWorks V8 software was employed to accomplish the spiral microfluidic system design. The platform was thought of as a multilayer assembly, working through the overlapping of ten carefully aligned square pieces with the patterns indicated in Figure 8. Each square has an edge length of 70 mm and one hole on each corner (i.e., 4 mm in diameter) to allow screw tightening of the final device. By overlapping the plates, channels are formed in such a way that allows a tangential flow of reagents and multiple mixing points within a single reaction chamber.

### 4.3. Microfluidic Platform Fabrication

**Figure 9:**
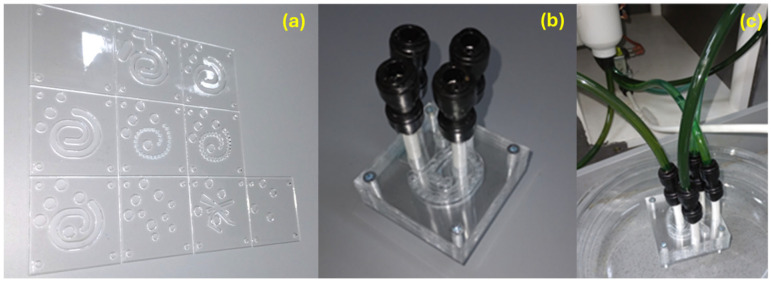
Microfluidic platform fabrication: (**a**) individual PMMA layers; (**b**) assembled overlayed microfluidic platform; and (**c**) microfluidic system integrated into the experimental setup.

The software-created designs were cut on 2 mm width PMMA sheets using a 1610 Pro laser cutting machine from RUBIQ CNC (Bacău, Romania). A 10 mm/s speed and 32% power were employed for cutting the patterns. The obtained plastic squares were further overlapped, aligned, and tightened together by screws (Figure 9). A thin layer of epoxy adhesive was spread on the edges of the platform and on the tubes entering the inlets and outlets of the system.

### 4.4. Fe_3_O_4_@SA Nanoparticle Synthesis

Nanoparticle synthesis involved the preparation of two solutions. The first solution was obtained by dissolving FeCl_3_ and FeSO_4_·7H_2_O in a 2:3 weight ratio in 900 mL of ultrapure water. The second solution was prepared by adding NaOH and salicylic acid to 900 mL of ultrapure water. Both solutions were simultaneously introduced in the microfluidic platform with the aid of a classical osmosis pump (PSP 220 Pump, Model No. CAR6003, Water Quality Association, Lisle, IL, USA; 90 mL/s flow rate). In more detail, iron precursor solution was passed through the microfluidic channels (round holes of 40 μm in diameter from layer 5), while the precipitation medium/functionalization solution was introduced through the two round orifices (8 mm diameter, also from layer 5), with both of the solutions meeting in the spiral mixing chamber across the two identical plates (layers 3 and 4). The resulting products were collected from the outlet channel of the chip (starting from layer 2 and exiting the platform at layer 10), magnetically separated, washed with ultrapure water several times, and dispersed using an ultrasonic processor (750 W; 50% pulse for 10 s/break for 3 s).

### 4.5. Nanoparticle Characterization

The microfluidic-synthesized magnetic material was characterized by a series of physicochemical and biological analyses.

X-ray Diffraction (XRD) analysis was employed to check the crystallinity of the Fe_3_O_4_@SA samples. For this investigation, a PANalytical Empyrean diffractometer (PANalytical, Almelo, The Netherlands), equipped with CuKα radiation source (λ = 1.5406 Å), was used at ambient temperature at a current of 40 mA and a voltage of 45 kV. Grazing incidence XRD scans were taken at an incidence angle of ω = 0.5°, for the Bragg angles (2θ) ranging between 20° and 70°.

The chemical composition of the dried magnetic powder was analyzed by Fourier-Transform Infrared Spectroscopy (FT-IR). The investigation was performed with a Thermo iN10-MX FTIR spectrometer (Thermo Fischer Scientific, Waltham, MA, USA) equipped with a ZnSe crystal. In total, 64 scans were collected in the range of 4000–400 cm^−1^, using a resolution of 4 cm^−1^. The scans were further co-added and processed through Omnic 8.2.0. (Thermo Fisher Scientific) software.

Information on the hydrodynamic diameter and zeta potential of the nanoparticles was obtained by Dynamic Light Scattering (DLS). For this investigation, we employed a DelsaMax Pro from Beckman Coulter (Brea, CA, USA), equipped with a 532 nm laser. The sample was prepared for analysis by dispersion in ultrapure water through ultrasonic treatment for 10 min. Then, the dispersion was injected into the measurement cell, and three acquisitions were made to ensure reliability (with the included data representing the average of these three measurements).

The morphology, size, and aggregation tendency of nanoparticles were investigated by Scanning Electron Microscopy (SEM) analyses. The SEM equipment also has an energy-dispersive spectroscopy (EDS) module, which was employed to determine sample elemental composition. A FEI Quanta Inspect F (Thermo Fisher—FEI, Eindhoven, The Netherlands) scanning electron microscope (SEM) equipped with a field emission gun (FEG) and a resolution of 1.2 nm was used for imaging morphology, size measurement, and aggregation tendency. The system also includes an energy-dispersive X-ray spectrometer (EDS) with a resolution of 133 eV at the MnKα line to determine the sample elemental composition.

More in-depth information was further gathered using Transmission Electron Microscopy (TEM) performed by a ThermoFisher Scientific 80–200 Titan Themis transmission electron microscope (Hillsboro, OR, USA). For the analysis, nanoparticles were dispersed in ethanol through 15 min of ultrasonic treatment. The sample was then placed onto a carbon-coated copper grid and dried at room temperature. TEM micrographs were taken at an accelerating voltage of 200 kV in transmission mode, employing point and line resolutions of 2 Å and 1 Å, respectively. Moreover, the utilization of the selected area electron diffraction (SAED) accessory allowed the obtaining of supplementary crystallographic data.

Two human cell lines were employed to assess the cytotoxicity of the nanoparticles synthesized using the newly developed microfluidic platform: MRC-5 cell line (lung fibroblasts) and HaCaT cell line (keratinocytes). Cells were cultured in Dulbecco’s Modified Eagle Medium (DMEM), supplemented with 10% fetal bovine serum (FBS) and 1% Penicillin/Streptomycin (P/S), under standard culture conditions (37 °C, 5% CO_2_, 95% humidity). To initiate the biological assays, cells were detached using trypsin/EDTA and seeded in sterile 96-well plates at an initial density of 1x10^4^ cells/well. After overnight incubation to ensure cell attachment, cells were exposed to various concentrations of Fe_3_O_4_@SA nanoparticles synthesized via the newly developed microfluidic platform. The nanoparticle concentrations subjected to the cytotoxicity screening were 1.22 mg/mL, 610 μg/mL, 305 μg/mL, 152.5 μg/mL, 76.25 μg/mL, and 38.125 μg/mL, prepared by serial dilutions of the stock solution in complete culture medium. For the experimental control (untreated cells), the cell culture medium was refreshed at the time of treatment initiation.

After 48 h of treatment, nanoparticle cytotoxic potential was evaluated using two complementary assays: the MTT assay to assess cell metabolic activity, and the Live/Dead viability assay to visualize live and dead cells and their distribution simultaneously.

For the MTT assay, the treatment medium was discarded and replaced with a 1 mg/mL solution of 3-(4,5-dimethilthiazol-2-il)-2,5-dipheniltetrazolium (MTT) in serum-free DMEM. Plates were incubated for 4 h at 37 °C to allow formazan crystal formation. Subsequently, the formazan crystals were solubilized in 2-propanol, and absorbance was measured at 550 nm using a FlexStation III multimodal reader (Molecular Devices, San Jose, CA, USA). Cell viability was expressed as a percentage relative to the untreated control, considered to be 100%. Data represents the mean ± standard deviation (SD) of three independent experiments. Statistical analysis was performed using GraphPad Prism software (10.5.0), with significance set as *p* ≤0.05.

For the Live/Dead assay, cell monolayers were rinsed with phosphate-buffered saline (PBS) following medium removal and incubated with a labeling solution containing calcein AM and ethidium bromide, as recommended by the manufacturer (LIVE/DEAD Viability/Cytotoxicity Kit). Staining was performed for 15 min at room temperature in the dark. After incubation, the labeling solution was removed, cells were washed with PBS, and imaging was performed using an Olympus IX73 fluorescent microscope equipped with Cell Sense F software V 8.0.2. Image analysis was conducted using ImageJ V 1.53.

## 5. Conclusions

This study introduces a novel 3D spiral microfluidic platform capable of the simultaneous synthesis and surface functionalization of Fe_3_O_4_ nanoparticles with salicylic acid in a continuous and reproducible manner. The multilayer chip design enabled efficient mixing, rapid reaction kinetics, and the formation of uniform, stable, and monodisperse magnetic nanostructures. A comprehensive physicochemical characterization confirmed the synthesized nanomaterials’ crystallinity, morphology, and surface chemistry. In addition, the results of the in vitro biological assays demonstrated a clear concentration-dependent response, highlighting the safety threshold below which Fe_3_O_4_@SA nanoparticles exhibit excellent biocompatibility toward human lung fibroblasts and keratinocytes as cellular models. These findings suggest that, with an appropriate dose optimization, Fe_3_O_4_@SA nanoparticles could represent a safe and versatile platform for biomedical applications. The chip’s modular design and operational simplicity also position it as a scalable and eco-friendly alternative to conventional nanoparticle synthesis methods. Additionally, future optimization will include an evaluation of synthesis reproducibility across multiple chip batches and extended production runs. Future work will focus on integrating this system with drug-loading capabilities and testing its functionality in more complex biological models.

## Figures and Tables

**Table 1:** Characteristics of synthesized Fe_3_O_4_@SA nanoparticles determined by DLS analyses.

Average Hydrodynamic Diameter (nm)	Standard Deviation	Zeta Potential (mV)	Standard Deviation	Polydispersity Index
146.28	0.38	78.544	1.26	0.2

**Table 2:** Comparison of key characteristics between traditional batch synthesis, 2D microfluidics, and the developed 3D spiral microfluidic platform.

Feature	Traditional Batch Synthesis	2D Microfluidics	3D Microfluidics (This Study)
Mixing efficiency	Low	Moderate	High (enhanced by 3D spiral flow)
Size control/monodispersity	Poor to moderate	Good	Excellent
Material consumption	High	Low	Low
Scalability	Moderate	Limited	Modular and stackable
Complexity/fabrication	Low	Low to moderate	Moderate to high
Functionalization integration	Post-synthesis	Limited	On-chip, simultaneous

## Data Availability

The original contributions presented in this study are included in the article. Further inquiries can be directed to the corresponding author.
